# Cascade Fluorescent Sensors Based on Isothermal Signal Amplification for the Detection of Mercury and Silver Ions

**DOI:** 10.3390/bios15040213

**Published:** 2025-03-26

**Authors:** Zhen Liu, Xing Liu, Jie Sun, Xilin Xiao

**Affiliations:** 1 School of Public Health, Hengyang School of Medicine, University of South China, Hengyang 421001, China; 20212014211130@stu.usc.edu.cn (Z.L.); 20241010110040@stu.usc.edu.cn (X.L.); 20232014211176@stu.usc.edu.cn (J.S.); 2 State Key Laboratory of Chemo/Biosensing and Chemometrics, Hunan University, Changsha 410082, China; 3 Department of Public Health Laboratory Science, University of South China, Hengyang 421001, China

**Keywords:** fluorescent sensor, mercury ion detection, silver ion detection, cascade isothermal signal amplification, catalytic hairpin assembly

## Abstract

In this study, novel fluorescent DNA biosensors for mercury (Hg^2+^) and silver (Ag^+^) ions were developed based on thymine (T)- and cytosine (C)-rich recognition elements in combination with exonuclease III and a mismatch-catalyzed hairpin assembly (MCHA)-based cascade isothermal signal-amplification strategy. In the presence of the respective target analytes, the recognition element terminals form so-called T-Hg^2+^-T or C-Ag^+^-C structures, resulting in cleavage by Exo III and the release of the trigger strand for MCHA. This binds to the H1 hairpin, which is fluorescently labeled with carboxyfluorescein (FAM) and tetramethylrhodamine (TAMRA), disrupting fluorescence resonance energy transfer between them and, thus, restoring FAM fluorescence, generating a strong signal at 520 nm. The linear range of the Hg^2+^ sensor is 0.5 to 3 pM, with a detection limit of 0.07 pM. The recovery range in actual spiked water samples is between 98.5% and 105.2%, with a relative standard deviation (RSD) ranging from 2.0% to 4.2%. The linear range of the Ag^+^ sensor is 10 to 90 pM, with a detection limit of 7.6 pM. The recovery range in actual spiked water samples is between 96.2% and 104.1%, with an RSD ranging from 3.2% to 6.3%. The cascade isothermal signal amplification strategy effectively enhances sensor sensitivity, while MCHA decreases the false-positive rate. The aptamer sensor exhibits high specificity, is resistant to interference, and can be used for the detection of Hg^2+^ and Ag^+^ in environmental water samples.

## 1. Introduction

Mercury ions (Hg^2+^) are environmentally persistent pollutants that bioaccumulate and enter the environment through various human activities, including—but not limited to—agricultural practices and pharmaceutical manufacturing [1]. They exhibit neurotoxic, genotoxic, and immunotoxic effects [2], making mercury pollution a globally important issue. Silver ions (Ag^+^) are widely used in photography, electronics, semiconductors, and the pharmaceutical industry, and they are widely distributed in the environment and food [3]. Nevertheless, Ag^+^ species are some of the most harmful metal pollutants. They exert toxic effects by affecting cellular processes in human gingival fibroblasts, keratinocytes, mast cells, and endothelial cells [4,5,6]. Additionally, Ag^+^ readily binds to enzymes and other proteins, leading to their inactivation [7]. Accordingly, significant research effort has been dedicated to developing low-cost, sensitive, selective, efficient, rapid, and flexible methods to quantitatively determine Hg^2+^ and Ag^+^ in natural and biological environments.

Traditional methods for detecting trace Hg^2+^ and Ag^+^ include the use of ion-selective electrodes [8], atomic absorption spectroscopy [9], surface-enhanced Raman scattering [10], hydride-generation atomic fluorescence spectrometry [11], and inductively coupled plasma mass spectrometry [12]. Although these detection techniques are highly accurate and reliable, their dependence on expensive equipment, complex sample-preparation procedures, and highly skilled operators greatly limits their application to rapid and on-site detection scenarios. Therefore, there is an urgent need to develop a method for Hg^2+^ and Ag^+^ detection that is both rapid and simple.

Thymine (T)-rich oligonucleotides can form mismatched base pairs in the presence of Hg^2+^ through the so-called T-Hg^2+^-T structure. Miyake et al. [13] reported that this structure is more stable than the Watson–Crick A–T base pair. Leveraging this capability, T-rich nucleic acid chains have been used extensively as recognition elements [14]. Various T-Hg^2+^-T-based biosensors have been developed for efficient and sensitive quantitative detection of Hg^2+^, such as those combined with surface-enhanced Raman scattering by Yao et al. [15], fluorescence by Jin et al. [16], colorimetry by Chen et al. [17], fluorescence resonance energy transfer by Hou et al. [18], and electrochemistry by Zhang et al. [19].

Similarly, Ag^+^ can interact selectively with cytosine (C) bases to form a stable C-Ag^+^-C structure, which converts single-stranded DNA into a more stable double-helix structure [20]. This unique interaction between C and Ag^+^ has garnered significant attention owing to its exceptional selectivity. The C–C mismatch shows a distinctive affinity for Ag^+^ [21]. Various forms of Ag^+^ biosensors have been developed based on this principle, including colorimetric biosensors [22] and electrochemical biosensors [23]. However, fluorescence-based biosensors have attracted particular attention owing to their convenient operation, rapid detection time, and high sensitivity [24].

The above two fluorescent biosensors, which rely on specific mismatched structures, are widely used in various detection fields due to their high sensitivity, rapid detection capabilities, suitability for high-throughput analysis, quick response, and high stability [25]. To further enhance the sensitivity of such biosensors, signal-amplification strategies are often incorporated.

Isothermal signal amplification, due to its simple experimental operation, constant reaction temperature, rapidity, and effectiveness, is comparable to the polymerase chain reaction technique, which is considered the gold standard. The isothermal signal amplification strategies currently used in fluorescence biosensors for heavy-metal-ion detection can be largely divided into enzyme-based, enzyme-free, and cascade types. Among these, enzyme-free isothermal signal amplification strategies include catalyzed hairpin assembly (CHA) [26], hybridization chain reaction (HCR) [27], and 3D Walkers [28]. CHA is particularly noted for its powerful amplification capability, simple protocol, enzyme-free and isothermal conditions, and versatility in combining with various signal-output modes for biosensing different analytes. In traditional CHA, H1, and H2 can bind nonspecifically due to environmental influences, even in the absence of an initiator, resulting in non-negligible background interference [29]. Therefore, introducing appropriate internal base pair mismatches at the hairpin tail end in CHA, termed mismatch CHA (MCHA), not only avoids false-positive signals, but also significantly enhances the reaction rate and amplification efficiency.

Exonuclease III (Exo III) does not require recognition of specific nucleic acid sequences to exert its enzymatic activity. It can continuously cleave from the blunt ends or 3′- recessed ends of double-stranded DNA (dsDNA), but it is inactive towards the 3′- ends of single-stranded DNA (ssDNA). Numerous studies have demonstrated that the enzymatic activity of Exo III can be stimulated by the formation of stable T-Hg^2+^-T complexes [30].

In this work, cascade fluorescence biosensors were constructed for the highly sensitive detection of Hg^2+^ and Ag^+^ using nucleic acid strands based on the mismatch structures T-Hg^2+^-T and C-Ag^+^-C, respectively, as recognition elements, combined with a signal amplification strategy based on Exo III and MCHA. Compared with single isothermal signal amplification strategies, cascade fluorescence biosensors exhibit higher sensitivity and specificity, along with a higher signal-to-noise ratio. Our results indicate that the developed biosensors achieve highly sensitive detection of Hg^2+^ and Ag^+^, and that they can be applied to the analysis of environmental water samples in the field.

## 2. Experimental

### 2.1. Instruments and Reagents

All chemicals and apparatus used in this paper are detailed in the Experimental section of the Supplementary Materials File S1.

### 2.2. Experimental Procedure

#### 2.2.1. Detection of Hg^2+^

First, we mixed 41 μL of buffer solution B with 3 μL of H0 (10.0 µmol·L^−1^) and 6 μL of Hg²⁺ solutions at various concentrations ranging from 0 mM to 100 mM. The mixture was reacted at 37 °C for 90 min. Then, to 20 μL of the reaction mixture was added 52 μL of buffer solution G and 3 μL of ExoⅢ(3U/μL), and the solution was incubated at 37 °C for 60 min for enzymatic digestion, followed by inactivation at 85 °C for 10 min. Finally, 219 μL of buffer C, 3 μL of H1 (4.0 µmol·L^−1^), and 3 μL of H2 (4.0 µmol·L^−1^) were added to the reaction system after inactivation treatment. The mixture was incubated at 37 °C for 2 h before measuring the fluorescence intensity.

#### 2.2.2. Detection of Ag^+^

First, we mixed 41 μL of buffer solution E with 3 μL of H’ (10.0 µmol·L^−1^) and 6 μL of Ag^+^ solutions at various concentrations ranging from 0 mM to 100mM. The mixture was reacted at 37 °C for 100 min. Then, to 20 μL of the reaction mixture was added 52 μL of buffer solution H and 3 μL of ExoⅢ (3U/μL), and the solution was incubated at 37 °C for 60 min for enzymatic digestion, followed by inactivation at 85 °C for 10 min. Finally, 219 μL of buffer F, 3 μL of H1 (4.0 µmol·L^−1^), and 3 μL of H2 (4.0 µmol·L^−1^) were added to the reaction system after inactivation treatment. The mixture was incubated at 37 °C for 2 h before measuring the fluorescence intensity.

#### 2.2.3. Fluorescence Determination

After the reaction was completed, the solution was scanned at λ_ex_ = 485 nm and the fluorescence intensity was measured at 520 nm (scan range: 500~650). All samples were analyzed in triplicate. Fluorescence detection was performed using a cuvette with a 1 cm path length, a slit width of 5 × 5 nm, a voltage of 700 V, and a scanning speed of 1200 nm/min.

#### 2.2.4. Polyacrylamide Gel Electrophoresis

The completion of the MCHA system reaction and the detection of Hg^2^⁺ and Ag⁺ were verified using polyacrylamide gel electrophoresis (PAGE). For 15% PAGE, 10 mL of 30% acrylamide, 5 mL of 5× TBE, 0.5 mL of 10% ammonium persulfate (freshly prepared), and 0.01 mL of N,N,N’,N’-tetramethylethylenediamine were added to 4.8 mL of ultrapure water and mixed quickly to form the gel. Different DNA samples were mixed with DNA loading buffer (5:1 volume ratio) and injected into different wells of the gel. Electrophoresis was conducted at 120 V for 60 min in 1× TBE (pH 8.2). Finally, the gel was stained with Goldview staining solution for 30 min and imaged using the ChemiDoc XRS system.

## 3. Results and Discussion

### 3.1. Detection Mechanism and Principle of the Sensors

**Scheme 1:**
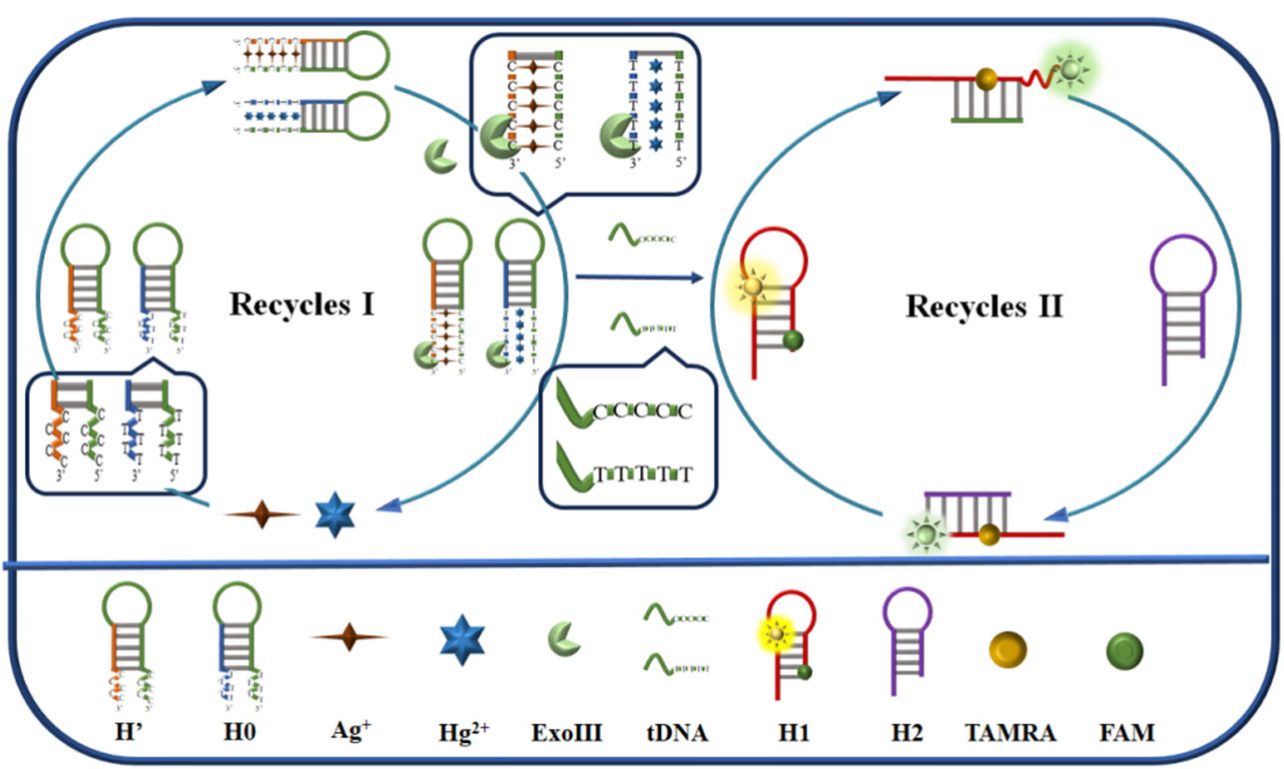
Illustration showing the principle of Hg^2^⁺ and Ag⁺ detection using a cascade-type fluorescent DNA sensor based on exonuclease III (Exo III) and mismatch-catalyzed hairpin assembly (MCHA).

The detection mechanism and principle of the cascade-type fluorescent DNA sensors are illustrated in Scheme 1. The recognition elements H0 and H’ can bind to Hg^2^⁺ and Ag⁺, respectively. In the absence of the target analytes, the three hairpin chains H0, H1, and H2 coexist stably in the Hg^2^⁺-detection system, while H’, H1, and H2 coexist stably in the Ag⁺-detection system. Therefore, the distance between the two fluorescent groups on the modified H1, i.e., carboxyfluorescein (FAM) and tetramethylrhodamine (TAMRA), remains unchanged, and under the action of fluorescence resonance energy transfer (FRET), FAM is quenched and TAMRA exhibits only partial fluorescence. In the presence of the target analytes, reactions occur in both systems, forming T-Hg^2^⁺-T with H0 and Hg^2^⁺, and C-Ag⁺-C with H’ and Ag⁺. Subsequently, H0 or H’ is specifically cleaved by Exo III, releasing Hg^2^⁺ or Ag⁺ and short DNA fragments (tDNA). The released analytes can bind to new recognition elements, completing the target analyte recognition cycle. The free tDNA acts as an auxiliary strand to trigger the subsequent MCHA reaction, generating the rigid H1–H2 double-stranded product, which significantly decreases the FRET effect, thus, restoring the fluorescence of FAM. The displaced tDNA serves as an auxiliary strand for the next round of MCHA, achieving effective signal amplification through two cycles. By measuring the fluorescence intensity of FAM, Hg^2^⁺, and Ag⁺ can be quantitatively determined.

### 3.2. Validation of Method Feasibility

**Figure 1:**
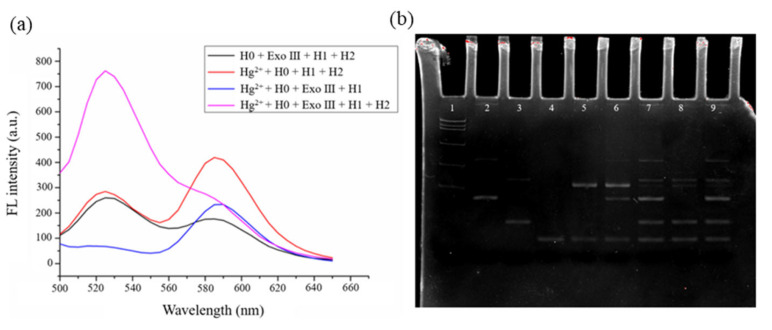
(**a**) Fluorescence spectra for the cascade-type fluorescent Hg^2^⁺ sensor. (**b**) Electrophoresis patterns of the cascade-type fluorescent Hg^2^⁺ sensor.

To examine the practical effectiveness of the sensors, fluorescence and PAGE analyses were performed for the four key steps of the Hg²⁺ and Ag⁺ reaction systems. The fluorescence spectrum in Figure 1a shows that in the absence of Hg²⁺, H2, or ExoⅢ, the system exhibits low fluorescence intensity at 520 nm. However, upon the introduction of Hg²⁺, the fluorescence intensity at 520 nm significantly increases, demonstrating the excellent responsiveness of the sensor to Hg²⁺ with a low background signal. Additionally, at 590 nm is the peak of tetramethylrhodamine. Only when the detection substance exists does the system undergo fluorescence resonance energy transfer, causing this peak to decrease or disappear. The electrophoresis patterns in Figure 1b (lanes 2 to 9) represent H0, H1, H2, H1 + H2 + tDNA, H0 + H1 + H2 + Hg²⁺ + ExoⅢ, H0 + H1 + H2 + Hg²⁺, H1 + H2 + Hg²⁺ + ExoⅢ, and H0 + H1 + H2 + ExoⅢ, respectively. The results for lanes 5 and 6 indicate that only when H0, H1, H2, ExoⅢ, and Hg²⁺ are present in the system can the MCHA reaction be triggered to produce the H1–H2 double-stranded product, proving the feasibility of this sensor for Hg²⁺ detection. 

**Figure 2:**
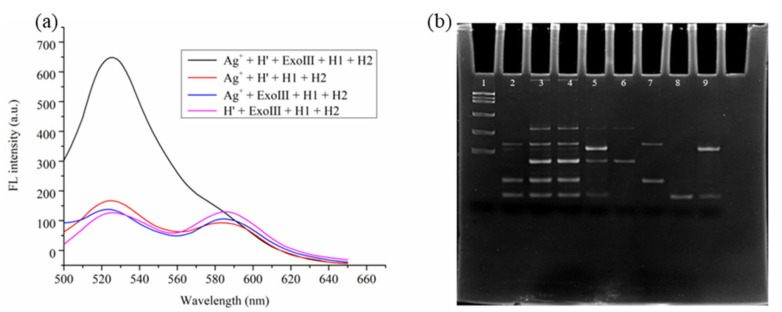
(**a**) Fluorescence spectra of the cascade-type fluorescent Ag⁺ sensor. (**b**) Electrophoresis results for the cascade-type fluorescent Ag⁺ sensor.

After replacing H0 with H’, this sensor system exhibits excellent responsiveness to Ag⁺, as shown by the fluorescence spectra in Figure 2a. The electrophoresis patterns in Figure 2b (lanes 2 to 9) represent H1 + H2 + Ag⁺ + ExoIII, H’ + H1 + H2 + ExoIII, H’ + H1 + H2 + Ag⁺, H’ + H1 + H2 + Ag⁺ + ExoIII, H’, H1, H2, and H1 + H2 + tDNA, respectively. The results for lanes 5 and 9 indicate that only when H’, H1, H2, ExoIII, and Ag⁺ are present in the system is the MCHA reaction triggered to produce the H1–H2 double-stranded product, proving the feasibility of this sensor for Ag⁺ detection.

### 3.3. Sensor Optimization

#### 3.3.1. Optimization of Conditions for the Hg^2^⁺ Sensor

To improve the detection performance of this sensor, the conditions for the first cycle reaction were optimized, including the reaction time, Na⁺ concentration in the buffer, Mg^2^⁺ concentration, pH, Exo III concentration, and digestion time in the enzymatic reaction.

**Figure 3:**
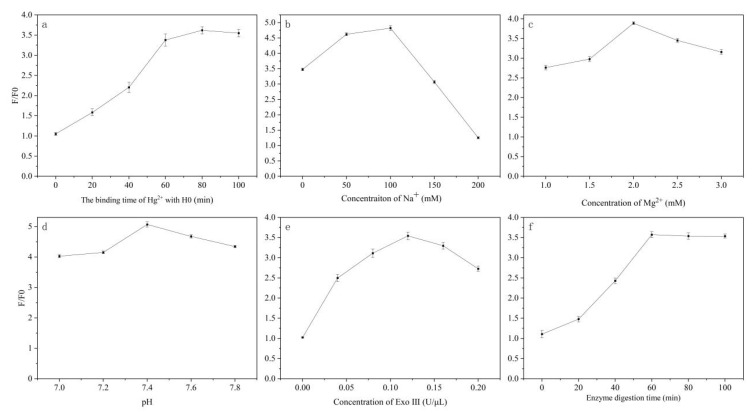
(**a**) Optimization of the binding time of Hg^2^⁺ with H0; (**b**) Optimization of Na⁺ concentration; (**c**) Optimization of Mg^2^⁺ concentration; (**d**) Optimization of pH; (**e**) Optimization of Exo III enzyme concentration; (**f**) Optimization of enzyme digestion time.

The reaction time between Hg^2^⁺ and H0 directly affects the amount of H0 that can be recognized by Exo III. Therefore, the binding time between Hg^2^⁺ and H0 was investigated first. As shown in Figure 3a, with the extension of reaction time, F/F0 gradually increased, reaching a maximum value at 80 min. Thus, 80 min was determined to be the optimal time for the binding of Hg^2^⁺ and the sensor probe.

Subsequently, the concentration of Na⁺, Mg^2^⁺, and the pH value in the buffer were optimized. Low Na⁺ concentrations can affect the stability of the DNA hairpin structure, resulting in a failure to correctly form or maintain the expected structure, thereby impacting the function of the recognition element and subsequent signal transduction. Conversely, excessively high Na⁺ concentrations can enhance the electrostatic interactions between DNA molecules, hindering the opening of hairpin DNA for specific binding with the analyte. As shown in Figure 3b, the F/F0 value reaches its peak at an Na⁺ concentration of 100 mM. Therefore, 100 mM was determined to be the optimal Na⁺ concentration in the buffer system.

Mg^2^⁺ can neutralize charge repulsion, enhancing the stability of the T-Hg^2^⁺-T structure and accelerating its formation. However, excessively high Mg^2^⁺ concentrations may lead to competition between Mg^2^⁺ and Hg^2^⁺ for binding sites, thereby affecting the detection results. As shown in Figure 3c, the F/F0 of the reaction system reaches its maximum when the Mg^2^⁺ concentration is 2 mM, thus the Mg^2^⁺ concentration was set to 2 mM.

When the pH value is too low or too high, the fluorescence performance of the fluorescent label is inhibited, resulting in a decrease in fluorescence signal intensity. As shown in Figure 3d, with increasing pH, F/F0 in this system initially increases and then decreases. F/F0 reaches its maximum value when the pH of the reaction system is 7.4, making 7.4 the ideal pH value for the buffer system.

Enzyme digestion is a crucial step to ensure the smooth progression of MCHA. Therefore, the concentration of Exo III and the digestion time were optimized. If the enzyme concentration is too low, the catalytic reaction rate decreases, reducing detection sensitivity. Conversely, if the enzyme concentration is too high, the reaction can become over-catalyzed and enter a supersaturated state, leading to interactions with nonspecific substrates or byproducts, affecting the accuracy and reproducibility of the results. As shown in Figure 3e, the effects of six different concentrations of Exo III on the reaction system were investigated. The F/F0 value peaked when the Exo III concentration was 0.12 U/μL, thus 0.12 U/μL was determined to be the optimal concentration for the enzyme digestion reaction.

If the enzyme digestion time is too short, the substrate does not fully react with the enzyme, resulting in incomplete reactions. If the enzyme digestion time is too long, side reactions may increase, causing reaction imbalance and affecting product stability. As shown in Figure 3f, the F/F0 value peaks when the digestion time reaches 60 min and then stabilizes, making 60 min the optimal digestion time.

#### 3.3.2. Optimization of Conditions for the Ag⁺ Sensor

Similarly to the Hg^2^⁺ fluorescence sensor, the conditions for the first cycle reaction were optimized, including reaction time, Na⁺ concentration in the buffer, Mg^2^⁺ concentration, pH, and Exo III concentration.

**Figure 4:**
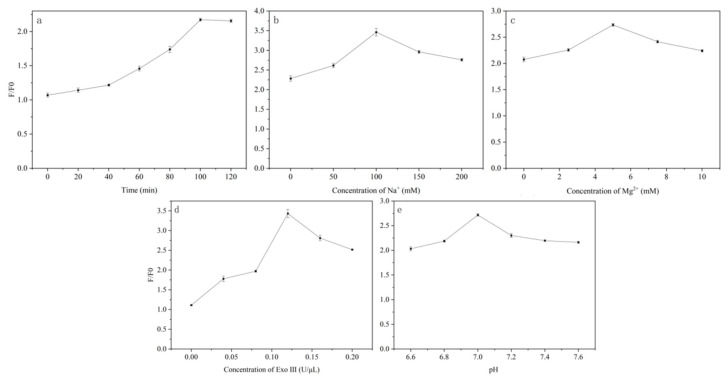
(**a**) Optimization of the binding time of Ag⁺ with H’; (**b**) Optimization of Na⁺ concentration; (**c**) Optimization of Mg^2^⁺ concentration; (**d**) Optimization of Exo III enzyme concentration; (**e**) Optimization of pH.

First, the binding time of Ag⁺ with H’ was investigated. As shown in Figure 4a, F/F0 gradually increases with the extension of the reaction time, reaching its highest value at 100 min. Therefore, 100 min was determined as the optimal binding time for Ag⁺ and H’.

Next, the concentrations of Na⁺ and Mg^2^⁺ in the buffer and the pH were optimized. As shown in Figure 4b, the F/F0 of the reaction system initially increases with Na⁺ concentration, peaks, and then decreases. F/F0 reached its maximum when the Na⁺ concentration was 100 mM, thus, 100 mM was determined as the optimal Na⁺ concentration in the buffer system. As shown in Figure 4c, F/F0 of the reaction system reached its maximum when the Mg^2^⁺ concentration was 5 mM, thus, 5 mM was set as the Mg^2^⁺ concentration. As shown in Figure 4d, F/F0 of the system exhibited an increasing and then decreasing trend with an increasing pH. F/F0 reached its maximum when the pH of the reaction system was 7.0, thus, 7.0 was set as the optimal pH value for the buffer system in the first step reaction.

Finally, the concentration of Exo III in the enzyme digestion reaction was investigated. As shown in Figure 4e, six different concentrations of Exo III were used to determine their effects on the reaction system. F/F0 peaked when the Exo III concentration reached 0.12 U/μL, thus, 0.12 U/μL was determined as the optimal Exo III concentration in the enzyme digestion reaction.

### 3.4. Sensor Performance

#### 3.4.1. Performance of the Hg^2^⁺ Sensor

**Figure 5:**
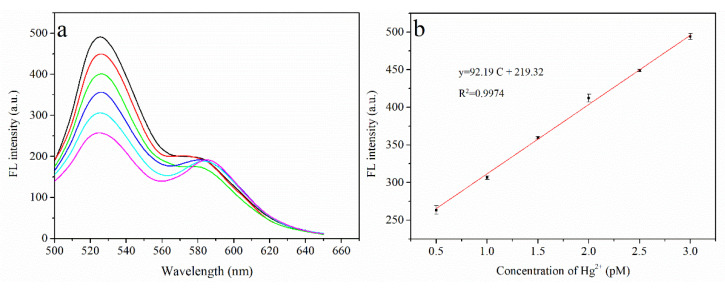
(**a**) Fluorescence spectra of the system, From bottom to top: fluorescence peaks of Hg^2+^ ions ranging from 0.5 pM to 3.0 pM at intervals of 0.5 pM; (**b**) Linear relationship of Hg^2+^ concentration with fluorescence intensity (0.5 pM to 3.0 pM).

With the optimized experimental conditions established, the performance of the sensor for Hg^2^⁺ detection was investigated. As shown in Figure 5a, the fluorescence intensity measured at 520 nm gradually increases with Hg^2^⁺ concentration (the Hg^2^⁺ concentration is plotted on the *x*-axis, and the fluorescence intensity at 520 nm is plotted on the *y*-axis). As depicted in Figure 5b, a good linear relationship is observed between the measured fluorescence intensity and Hg^2^⁺ concentration in the range 0.5 to 3 pM, with the linear equation y = 92.19C + 219.32 (where *R*^2^ = 0.9974). The detection limit of this system was calculated to be 0.07 pM.

#### 3.4.2. Performance of the Ag⁺ Sensor

**Figure 6:**
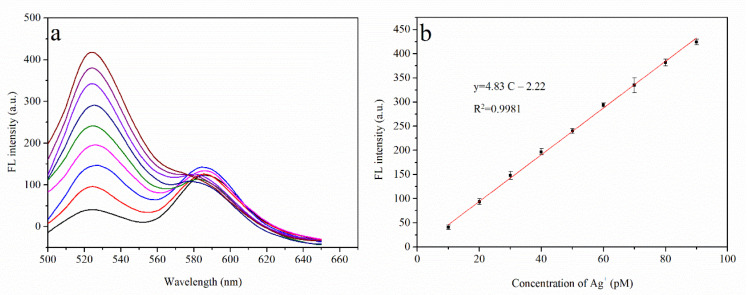
(**a**) Fluorescence spectra of the system. From bottom to top: fluorescence peaks of Ag⁺ ions ranging from 10 pM to 90 pM at intervals of 10 pM; (**b**) Linear relationship of Ag^+^ concentration with fluorescence intensity (10 pM to 90 pM).

With the optimized experimental conditions established, the performance of the sensor for Ag⁺ detection was investigated. As shown in Figure 6a, the fluorescence intensity measured at 520 nm gradually increases with Ag⁺ concentration (the Ag⁺ concentration is plotted on the *x*-axis, and the fluorescence intensity at 520 nm is plotted on the *y*-axis). As depicted in Figure 6b, a good linear relationship is observed between the measured fluorescence intensity and Ag⁺ concentration in the range 10 to 90 pM, with the linear equation y = 4.83C − 2.22 (where *R*^2^ = 0.9981). The detection limit of this system was calculated to be 7.6 pM.

### 3.5. Sensor Selectivity

#### 3.5.1. Selectivity of the Hg^2^⁺ Sensor

**Figure 7:**
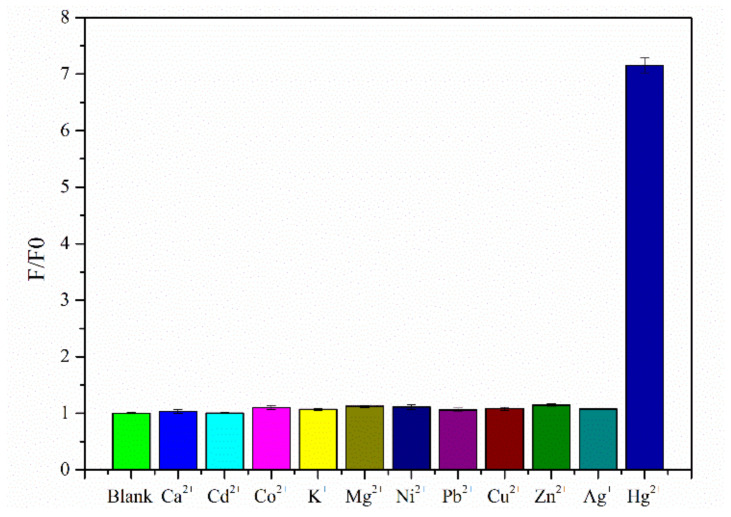
Selectivity results for the Hg^2^⁺ sensor.

To evaluate the specific recognition of Hg^2^⁺ by the detection system, 10 other metal ions were selected for analysis, including Ca^2^⁺, Cd^2^⁺, Co^2^⁺, K⁺, Mg^2^⁺, Ni^2^⁺, Pb^2^⁺, Cu^2^⁺, Zn^2^⁺, and Ag⁺. The concentration of the added metal ions was 50 times that of Hg^2^⁺. The response signals reflected by the fluorescence intensity at 520 nm were significantly lower compared with that for Hg^2^⁺ (Figure 7) indicating that the novel sensor has good selectivity for Hg^2^⁺.

#### 3.5.2. Selectivity of the Ag⁺ Sensor

**Figure 8:**
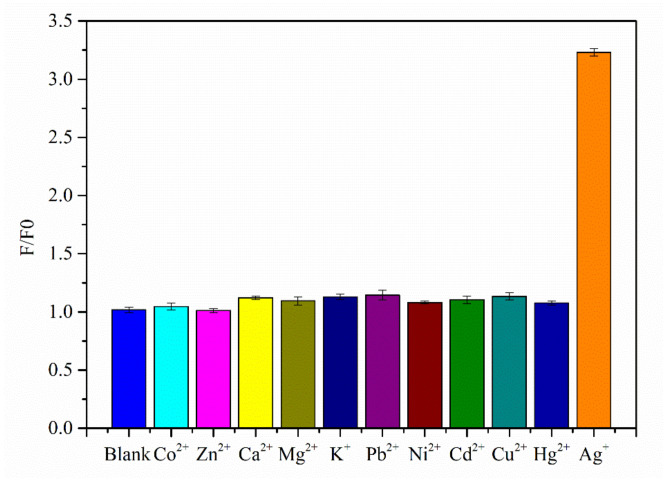
Selectivity results for the Ag⁺ sensor.

To evaluate the specific recognition of Ag⁺ by the detection system, 10 other metal ions were selected for analysis, including Co^2^⁺, Zn^2^⁺, Ca^2^⁺, Mg^2^⁺, K⁺, Pb^2^⁺, Ni^2^⁺, Cd^2^⁺, Cu^2^⁺, and Hg^2^⁺. The concentration of the added metal ions was 50 times that of Ag⁺. The response signals reflected by the fluorescence intensity at 520 nm are significantly lower compared with that for Ag⁺ (Figure 8), indicating that the novel sensor has good selectivity for Ag⁺.

### 3.6. Application to Real-World Samples

#### 3.6.1. Application of the Hg^2^⁺ Sensor

To evaluate the application capabilities of the Hg^2^⁺ and Ag⁺ sensors to real samples, water samples from the Xiangjiang River in Hengyang, Hunan, China, and tap water were selected for spiked recovery tests. The spiked recovery results for the Hg^2^⁺ cascade fluorescence sensor are shown in Table 1 (where the mean is the average of three test results, and SD is the standard deviation of three test results). To assess the reliability of this method, spiked water samples with three different concentrations of Hg^2^⁺ (1.2, 1.4, and 1.6 pM) were prepared for fluorescence detection. The recovery rates of Hg^2^⁺ from the spiked Xiangjiang water samples ranged from 98.53% to 105.18%, with RSDs between 2.0% and 4.2%. For the tap water samples, the recovery rates of spiked Hg^2^⁺ ranged from 101.9% to 104.3%, with RSDs between 3.3% and 4.1%. Therefore, the developed cascade fluorescence sensor can be applied for the detection of Hg^2^⁺ in water samples, demonstrating the feasibility of this method for actual detection scenarios.

#### 3.6.2. Application of the Ag⁺ Sensor

Table 2 shows the spiked recovery results for the Ag^+^ cascade fluorescent sensor. Three concentrations of Ag^+^ (30, 50, and 70 pM) were used in spiked water samples for fluorescence detection. The recovery rates of Ag^+^ in spiked Xiangjiang River water samples were between 96.2% and 104.1%, with RSD ranging from 3.2% to 6.3%. In the tap water samples, the spiked recovery rates of Ag^+^ were between 97.8% and 102.6%, with RSD ranging from 3.2% to 4.5%. Therefore, the developed cascade fluorescent sensor can be applied for the detection of Ag^+^ in water samples, demonstrating the feasibility of this method in practical detection.

### 3.7. Comparison with Reported Fluorescence Detection Systems

Lu et al. [31] employed ultrathin two-dimensional MXenes as a fluorescent quencher and a Hg^2^⁺-induced Exo III-assisted target recycling strategy for efficient signal amplification, constructing a rapid and sensitive fluorescent bioanalytical platform for Hg^2^⁺. Ravikumar et al. [32] reported a highly sensitive fluorescent biosensor for Hg^2^⁺ determination in aqueous solvents based on an Exo III-assisted signal amplification mechanism. Zhong et al. [33] developed a fluorescent sensor based on Exo III-assisted signal amplification combined with a triplex molecular switch. Li et al. [34] constructed a fluorescent sensor based on CHA and Mg^2^⁺-dependent DNAzymes. Chen et al. [35] developed a fluorescent biosensor based on CHA combined with microchip electrophoresis. As shown in Table 3, compared with other fluorescent sensors that use Exo III and CHA individually, the Hg^2^⁺ cascade fluorescent sensor constructed here has a lower limit of detection (LOD) and probability of false-positive results.

Wei et al. [36] developed a highly efficient and sensitive fluorescent sensing platform for detecting Ag^+^ based on the specific interaction between Ag^+^ and cytosine–cytosine (C–C) base mismatches, using berberine as the fluorescent probe and ExoI for the signal amplification strategy. Li et al. [21] proposed a fluorescent biosensor based on the C-Ag^+^-C structure with a novel combination design featuring Exo III dual-cycle amplification for Ag^+^ detection applications. Li et al. [37] developed a highly efficient and sensitive fluorescent Ag^+^ sensor based on a dual-signal amplification strategy using glucose oxidase and HCR. Chen et al. [35] combined Exo III and CHA for efficient signal amplification, constructing a fluorescent biosensor for the detection of Hg^2+^ and Ag^+^. As shown in Table 4, the Ag^+^ cascade-type fluorescent sensor constructed here has a lower LOD compared with other fluorescent sensors using Exo III and CHA separately.

## 4. Conclusions

In this study, we constructed a highly sensitive and selective cascade fluorescent biosensor using a combined MCHA and Exo III-mediated isothermal signal amplification strategy for the effective detection of Hg^2+^ and Ag^+^. In optimal experimental conditions, the fluorescence intensity of the Hg^2+^ cascade fluorescent sensor at λ_em_ = 520 nm increases linearly, with a correlation coefficient of 0.9974, a linear range of 0.5 to 3 pM, and a detection limit of 0.07 pM. The recovery range in actual spiked water samples was between 98.5% and 105.2%, with RSD between 2.0% and 4.2%, demonstrating the applicability of this method for the detection of Hg^2+^ in environmental water samples.

For the Ag^+^ cascade fluorescent sensor, the fluorescence intensity at λ_em_ = 520 nm also increases in optimal conditions, with a correlation coefficient of 0.9981, a linear range of 10 to 90 pM, and a detection limit of 7.6 pM. The recovery range in actual spiked water samples was between 96.2% and 104.1%, with RSD between 3.2% and 6.3%, proving the applicability of this method for the detection of Ag^+^ in environmental water samples.

The Exo III and MCHA-based cascade fluorescent DNA biosensor constructed in this study demonstrates excellent detection performance, providing an effective method for the highly sensitive detection of Hg^2+^ and Ag^+^. The cascade signal amplification strategy used significantly improves the sensitivity and specificity of the sensor. Furthermore, the introduction of the MCHA method effectively decreases the generation of false-positive signals.

We acknowledge that there are still deficiencies in this study. In subsequent re-search, we will conduct additional investigations on the interference of other factors, such as organic molecules, natural chelators, emerging contaminants, etc. Moreover, we will enhance the research on the performance of the biosensor under different temperature conditions and during long-term storage. Although this experiment did not directly explore the contrast potential of the probe, based on the current design, the emission peak of TAMRA (590 nm) and the signal recovery mechanism of FAM have already provided a good foundation for improving the probe’s contrast and sensitivity. The stable TAMRA signal can serve as an internal reference, offering strong support for signal analysis in practical applications, especially in complex sample environments. We will further explore this in future research. This approach shows great potential for the field of biosensors, and with continuous optimization and innovation it is expected to achieve more significant results in improving detection sensitivity and reducing false-positive rates, thus, providing strong support for practical applications and commercialization.

## Figures and Tables

**Table 1:** Detection of Hg^2+^ in water samples (*n* = 3).

Water Sample	Spiked Concentration (pM)	Measured Value (Mean ± SD)	RSD (%)	Recovery Rate (%)
Xiangjiang River Water sample 1	1.20	1.18 ± 0.05	4.2	98.5
Xiangjiang River Water sample 2	1.40	1.43 ± 0.03	2.0	102.1
Xiangjiang River Water sample 3	1.60	1.68 ± 0.03	2.0	105.2
Tap Water sample 1	1.20	1.21 ± 0.04	3.3	100.8
Tap Water sample 2	1.40	1.46 ± 0.06	4.1	104.3
Tap Water sample 3	1.60	1.63 ± 0.05	3.1	101.9

**Table 2:** Detection of Ag^+^ in water samples (*n* = 3).

Water Sample	Spiked Concentration (pM)	Measured Value (Mean ± SD)	RSD (%)	Recovery Rate (%)
Xiangjiang River 1	30.00	28.87 ± 1.83	6.3	96.2
Xiangjiang River 2	50.00	49.21 ± 1.66	3.4	98.4
Xiangjiang River 3	70.00	72.84 ± 3.35	4.6	104.1
Tap Water sample 1	30.00	29.56 ± 1.33	4.5	102.6
Tap Water sample 2	50.00	50.76 ± 2.14	4.2	97.8
Tap Water sample 3	70.00	71.64 ± 2.29	3.2	101.9

**Table 3:** Comparison of different fluorescence detection systems for Hg^2+^.

Type	Strategy	LOD	Linear range	Samples	Citation
Enzyme-based tools	ExoIII	42.5 pM	0.05–50 nM	Tap water and river water	[31]
	ExoIII	10 pM	0–0.1 nM	Tap water and river water	[32]
	ExoIII	1.04 pM	0.01–50 nM	Tap water and river water	[33]
Non-enzymatic	CHA	7.9 pM	10 pM–10 nM	Tap water and river water	[34]
	CHA	0.054 nM	0.18–50 nM	Tap water and river water	[35]
Cascade	ExoIII and MCHA	0.07 pM	0.5–3 pM	Xiangjiang River water and tap water	This work

**Table 4:** Comparison of different fluorescence detection systems for Ag^+^.

Type	Strategy	LOD	Linear Range	Samples	Citation
Enzyme-based tools	Exo I	4.4 nM	0.0059–235.48 μM	Tap water and serum	[36]
	Exo III	2 nM	5–1500 nM	Lake water	[21]
Non-enzymatic	HCR	1.8 nM	5–1500 nM	Lake water and tap water	[37]
Cascade	Exo III and CHA	0.038 nM	0.12–100 nM	Water samples and milk	[35]
	Exo III and MCHA	7.6 pM	10–90 pM	Xiangjiang River water and tap water	This work

## Data Availability

Data sharing is not applicable to this article as no new data were created or analyzed in this study.

## Supplementary Materials

The following supporting information can be downloaded at: https://www.mdpi.com/article/10.3390/bios15040213/s1, more details in the Supplementary Materials File S1.
