# Supplementary material for: Cascade Fluorescent Sensors Based on Isothermal Signal Amplification for the Detection of Mercury and Silver Ions
Source: Biosensors (Basel). 2025 Mar 26;15(4):213. doi: 10.3390/bios15040213 (PMC12025150; doi:10.3390/bios15040213)
Supplement: Supplementary file 1 [file biosensors-15-00213-s001.zip › biosensors-3508067-supplementary.pdf]

biosensors

MDPI

Supplementary

Cascade Fluorescent Sensors Based on Isothermal Signal Amplification for the Detection of Mercury and Silver Ions

Chemicals. Exonuclease III and Ne Buffer were purchased from Shanghai Jinpan Biotechnology Co., Ltd. (Shanghai, China). Mercuric nitrate, anhydrous magnesium chloride, sodium chloride, silver nitrate, disodium hydrogen phosphate, and dithiothreitol (DTT) were obtained from Shanghai Aladdin Biochemical Technology Co., Ltd. (Shanghai, China). Magnesium nitrate was sourced from Guangzhou Chemical Reagent Factory (Guangzhou, China). Sodium nitrate and nitric acid were acquired from Guangdong Chemical Reagent Engineering Research and Development Center (Guangzhou, China). Tris(hydroxymethyl)aminomethane was obtained from Tianjin Damao Chemical Reagent Factory (Tianjin, China). Hydrochloric acid was purchased from Hunan Shaoyang Chemical Reagent Factory (Shaoyang, China). 30% Acr-Bis, TBE and 6×DNA Loading buffer were obtained from Wuhan Seville Biotechnology Co., Ltd. (Wuhan, China). Ammonium persulfate was sourced from Aladdin Reagent Co., Ltd. (Shanghai, China). Goldview was sourced from Fuzhou Feijing Biotechnology Co., Ltd. (FuZhou China). Thermo Scientific Pierce (Temed) was sourced from Thermo Fisher Scientific Co., Ltd. (Waltham, Massachusetts, United States). All reagents were of analytical grade or higher purity. Ultrapure water (≥18 MΩcm) was used for all experiments.

Nucleic Acid Strands. H0: 5′-TTTTTTGGTAGAAAAGGTCACTCACTATAAACCCATAAATAGTGAGTGACCTTTTCTACCTTTTTT-3′. H′: CCCCCCGGTAGAAAAGGTCACTCACTATAAACCCATAAATAGTGAGTGACCTTTTCTACCCCCCCC. H1: ATAGTGAGTGACCT(TAMRA)TTTCTACCACCAGACTACGGTAGAAAAGGT(FAM)CACTGT. H2: TCTACCGTAGTCTGGTGGTAGAAAAGGAACCAGACTAC. All nucleic acid strands were 2× HPLC purified and purchased from Sangon Biotech Co., Ltd. (Shanghai, China).

Apparatus. Fluorescence spectrophotometer (Hitachi F-7000, Hitachi Ltd., Japan); pH meter (PHS-3, Shanghai INESA Scientific Instrument Co., Ltd., China); thermostatic water bath (DF-101S, Xicheng Xinrui Instrument Factory, Jintan District, Changzhou, Jiangsu, China); electronic balance (FA2004, Shanghai Hengping Scientific Instruments Co., Ltd., China); gel electrophoresis apparatus (DYCZ-24, Beijing Liuyi Biotechnology Co., Ltd., China); ChemiDoc XRS imaging system (DocTM XR+1708195, Bio-Rad Laboratories, USA).

Buffer Preparation. Buffer solution A (50 mM Na_2HPO_4, 500 mM NaCl, pH 7.6) was used for the annealing of H0, H1, and H2; Buffer B (10 mM Tris-HCl, 100 mM NaCl, 2 mM $MgCl_2$, pH 7.4) was used for the reaction of H0 with Hg^{2+}; Buffer C (10 mM Tris-HCl, 100 mM NaCl, pH 7.6) was used for the MCHA reaction in Hg^{2+} detection. Buffer solution D (50 mM Tris-HNO_3, 100 mM $NaNO_3$, 5 mM $Mg(NO_3)_2$, pH 7.0) was used for the annealing of H′, H1, and H2; Buffer E (10 mM Tris-HNO_3, 100 mM $NaNO_3$, 5 mM $Mg(NO_3)_2$, pH 7.4) was used for the reaction of H′ with Ag^+; Buffer F (10 mM Tris-HNO_3, 100 mM $NaNO_3$, 5 mM $Mg(NO_3)_2$, pH 7.6) was used for the MCHA reaction in Ag^+ detection. The prepared buffer solutions were sterilized at 121℃ and 0.1 MPa for 20 minutes, then cooled and stored at 4℃ for future use. Buffer G (NE Buffer) was used for the enzymatic reaction process in Hg^{2+} detection, and Buffer H (10 mM Tris-HNO_3, 10 mM $Mg(NO_3)_2$, 1 mM DTT, pH 7.0) was used for the enzymatic reaction process in Ag^+ detection, and stored at -20℃ for future use.

Preparation of DNA Stock Solutions. The lyophilized DNA powder was centrifuged for 1 minute before use to prevent trace amounts from adhering to the walls. H0, H1, and H2 were dissolved in buffer solution A, and H′, H1, and H2 were dissolved in

buffer solution D, to prepare 10 μM stock solutions. The solutions were heated at 95℃ for 5 to 10 minutes, then slowly cooled to room temperature, and stored at 4℃ for future use.
